# Clinical profile and outcome of pediatric heart surgeries at semi-urban tertiary care cardiac center of Gujarat, India: a 54-month single-center retrospective chart review

**DOI:** 10.3389/fped.2025.1543755

**Published:** 2025-05-20

**Authors:** Rahul Tandon, Vishal V. Bhende, Amit Kumar, Naresh Dhedhi, Mamta R. Patel, Krutika Rahul Tandon

**Affiliations:** ^1^ Department of Pediatrics, Pramukhswami Medical College, Bhaikaka University, Karamsad, India; ^2^ Department of Pediatric Cardiac Surgery, Bhanubhai and Madhuben Patel Cardiac Centre, Shree Krishna Hospital, Karamsad, India; ^3^ Central Research Services, Bhaikaka University, Karamsad, India

**Keywords:** acyanotic, congenital heart disease, cyanotic, intracardiac repair, pulmonary artery/arterial hypertension, redo surgery, surgery, ventricular dysfunction

## Abstract

**Context:**

The most common congenital condition, congenital heart disease (CHD), is often left untreated or doesn't get timely attention for various reasons. Nowadays, many pediatric cardiac centers in India offer surgical management, even in semi-urban or rural areas. Our center is one of them.

**Aims:**

This study aims to generate regional data from the Western part of India, primarily to determine the outcome of patients who had surgical intervention at our pediatric cardiac center.

**Methods and material:**

The present study retrospectively analyzed the data of the operated pediatric patients by reviewing the charts of patients admitted to the pediatric division of the cardiac center between April 2018 and October 2022. All demographic details, anthropometry, preoperative evaluation, operative procedures, postoperative issues, outcomes, etc. were reviewed, and the collected data were entered into Microsoft Excel.

**Statistical analysis:**

Descriptive statistics were used to present demographic data. Chi-square and Fisher exact tests were applied to find an association, and a *p*-value of <0.05 was considered statistically significant.

**Results:**

Out of the 422 pediatric admissions during the period studied, 386 underwent cardiac surgeries. The median (Q1, Q3) age of patients in months was 12 (6, 60), and 251 (61.2%) were males. The most prevalent CHD was ventricular septal defect (VSD), accounting for 88 patients (21.6%). Out of the 386 total patients who underwent cardiac surgery, 16 (4.1%) patients experienced mortality. The most common surgeries performed were VSD closure, patent ductus arteriosus ligation, and intracardiac repair for tetralogy of Fallot (TOF).

**Conclusions:**

Pediatric heart surgery was offered for a varied CHD with comparable mortality and morbidity with other centers.

## Introduction

A status report on congenital heart disease (CHD) in India highlights that CHD is the most prevalent congenital condition, comprising 28% of all congenital birth defects, and represents a global health concern for children (1). An estimated 1.35 million children are diagnosed with CHD each year worldwide, with approximately 240,000 cases in India, reflecting a prevalence ratio of 9/1,000 (1). The same author noted in her 2005 status report that although resources are scarce and the scope of CHD is immense, its significance is growing because it is a preventable cause of death (2). However, due to multiple reasons, many patients remain untreated despite timely diagnosis. The diagnostic and treatment approaches may differ in resource-limiting settings in developed countries. In lower-middle-income countries, surgery should be made available, and its improvement is possible only by proper networking that allows early detection and referral to pediatric cardiac centers tailored to manage such problems. Here, pediatricians, obstetricians, and cardiologists play a vital role in implementing such a strategy (3, 4). CHD ranks seventh in terms of infant mortality and is the leading cause of death related to birth defects (5). Although the large gap prevails, the ray of hope is a program like “Hridyam,” which was implemented successfully in Kerala, India. In the Indian context, “Hridyam” is about the heart. Through various government schemes, management was offered to congenital cardiac defects. It was a population-based program that had a web-based application enabling real-time case registration, treatment referrals on a priority basis, and tracking of every child registered with CHD. Thus, through a public–private partnership, advanced pediatric heart care was provided. It had registered 502 cases in 2 years with a 30-day mortality of 2.4%. By implementing this program, Kerala reduced overall and CHD-related infant mortality by 21.1% and 41.0%, respectively. Therefore, it is proposed that the coming years, i.e., 2022–2047, will be a golden period if the same strategy is applied countrywide (6). In the Indian context, it will be “Amrit (Nectar—the drink of the god symbolizing immortality in Hindu mythology) Kaal (the Period)” (6). Although our region has lacked such a system, this single center with one surgeon has still managed roughly 520 cases in the last 7 years, which accounted for 70–75 cases per year or 6 cases per month. This study aimed to determine the outcome and postoperative results for pediatric patients admitted to our region's sole referral hospital.

## Subjects and methods

After institutional ethics committee approval with a waiver of consent, the present study retrospectively analyzed the data of the operated pediatric patients by reviewing the charts of patients admitted to the pediatric division of the cardiac center (Bhanubhai and Madhuben Patel Cardiac Center, Shree Krishna Hospital, Karamsad) between April 2018 and October 2022. This study was conducted at a tertiary care center that receives referrals from the entire region of Gujarat. Our center has advanced cardiac imaging, mechanical ventilator, and cardiac catheterization capabilities. We have a single pediatric cardiothoracic surgeon who can handle the entire spectrum of cases requiring cardiopulmonary bypass for open heart surgeries. However, patients who need extracorporeal membrane oxygenation (ECMO) require referral to an additional center. Postoperative cardiac patients are cared for in a dedicated cardiac PICU staffed by a 24/7 pediatric cardiac intensivist. In this study, we documented the mortality rates of patients who underwent surgery at our center. In addition, we recorded other details such as age, gender, distance of residence from our center, symptoms, weight, height, type of cardiac lesion, investigation modalities used for diagnosis, name of operation, and postoperative issues including ventricular dysfunction, pulmonary artery/arterial hypertension, or residual lesions. All data were retrieved from hospital records and entered into Microsoft Excel, followed by analysis using the STATA 14.2 version.

### Statistical analysis

Descriptive statistics (mean, standard deviation, median, interquartile range, minimum, maximum value, frequency, percentage, and proportion) were used to describe baseline characteristics. We used Fisher exact and Chi-square tests to evaluate for an association between lesion and demographics with mortality, and a *p*-value of <0.05 was considered statistically significant.

## Results

**Figure 1:**
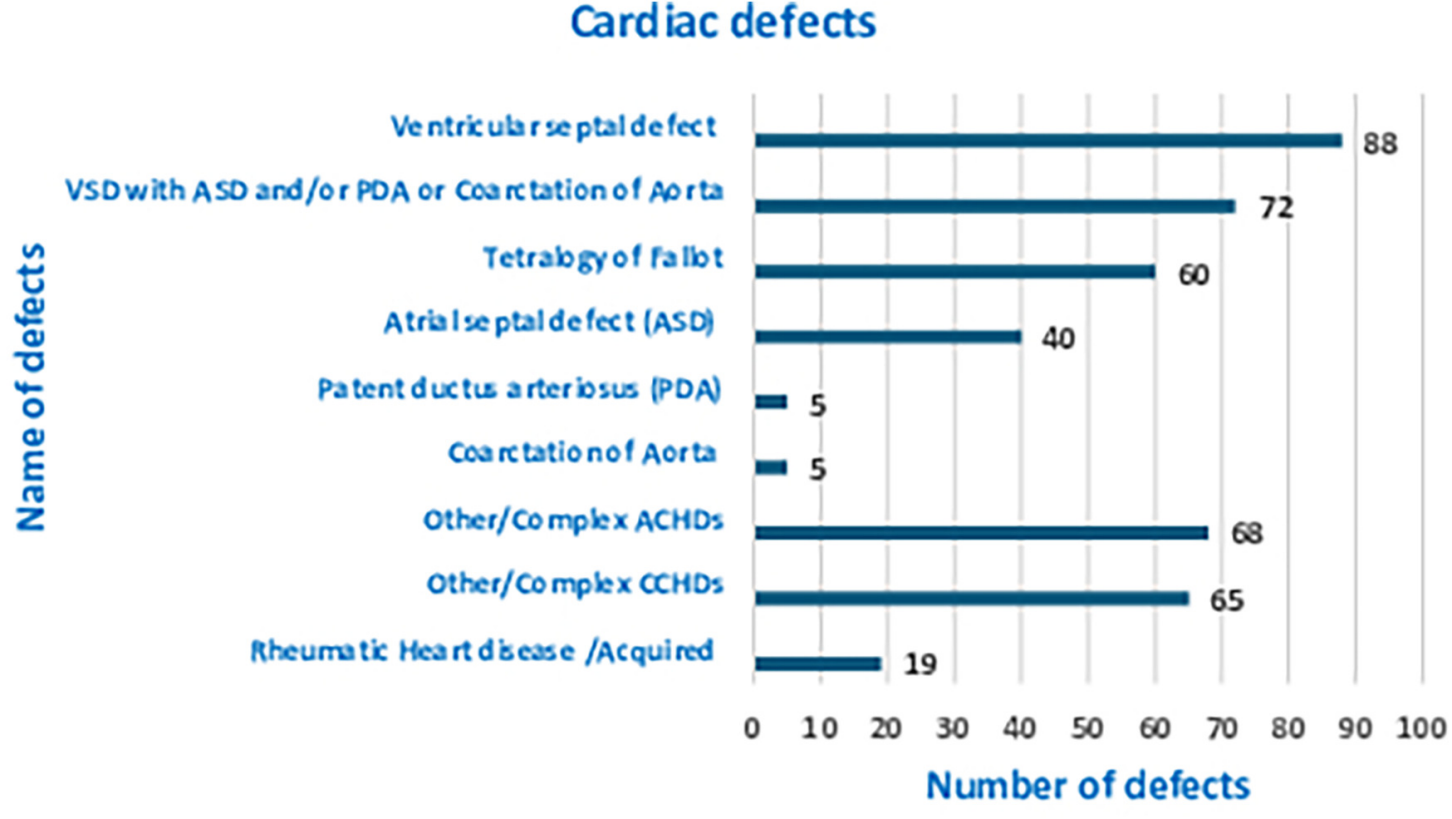
Graph of frequency and proportion of cardiac defects.

Out of the 422 admissions during the period studied, 386 underwent surgery. The median (Q1, Q3) (min, max) age of patients in months was 12 (6, 60) (1, 252), and 262 (62%) were males. A total of 16 (3.79%) patients died, but the males contributed to 93.33% mortality, and the infancy period contributed 40%. All baseline details are presented in Tables 1–3. The proportion of various cardiac defects is shown in Figure 1. Failure to gain weight/poor weight gain, recurrent respiratory tract infection, chest pain, or dyspnea on exertion cyanotic spells were the main clinical features in the present study cohort (Table 2). In the current study, acyanotic and cyanotic cardiac defects accounted for 278 (68.6%) and 125 (31.4%), respectively, wherein the commonest defect was ventricular septal defect (VSD)—21.84% (88/403) among acyanotic and tetralogy of Fallot's (TOF) and 14.89% (60/403) among cyanotic CHD variety. Table 4 shows the frequency of all performed cardiac operations/procedures, whereas Table 5 shows the mortality by operative procedures. We performed surgery on 138 patients with isolated acyanotic defects, 140 with other acyanotic defects, 68 with TOF, and 65 with complex cyanotic CHDs. In this cohort, 38.6% (*n* = 149) of patients experienced at least one postoperative issue. These included residual lesions (35.3%), ventricular dysfunction (right ventricular 7.3%, left ventricular 6.1%, biventricular 3.5%), redo surgery (5.2%), and pulmonary artery/arterial hypertension (30.6%). A total of 406 patients survived to discharge. There was 4.1% mortality (*n* = 16) among operated cases. Of those patients who died, five had VSD. Mortality was higher for males (*p* = 0.01) and those who experienced symptoms (*p* = 0.001). Patients with isolated acyanotic defects were most likely to experience LV dysfunction. An association of age, gender, and symptoms with outcome is shown in Table 6. The association of postoperative issues with types of cardiac defects is shown in Table 7. The median (Q1, Q3) (min, max) length of stay in hospital was 10 (7, 15.5) (1, 107) days.

**Table 1:** Demographic details of all patients (*n* = 422).

Variables	Category	*n* (%)
Gender	Male	262 (62)
	Female	160 (38)
Age in months	<12	233 (55)
	13–60	90 (21)
	61–120	63 (15)
	>120	36 (09)
Is the heart defect part of the syndrome?	Yes	13 (3.1)
	No	409 (96.9)
Residence	Own district	185 (43.84)
	Other districts of the state	221 (52.37)
	Outside the own state	13 (3.08)
	Outside own country	03 (0.71)

**Table 2:** Type and symptomatology of all patients (*n* = 422).

Variables	Category	*n* (%)
The broad category of cardiac defects	Congenital	403 (95.5)
	Acquired	19 (4.5)
The broad division of congenital defects (*n* = 403)	Acyanotic	278 (68.6)
	Cyanotic	125 (31.4)
Symptomatology at admission	Asymptomatic or non-specific symptoms or 2D echo screened	331 (78.4)
	Failure to thrive or poor weight gain	47 (11.14)
	Recurrent respiratory tract infection	22 (5.21)
	Cyanotic spell	12 (2.84)
	Chest pain or dyspnea on exertion	10 (2.37)

**Table 3:** Usage of diagnostic modalities, postoperative issues, and outcome.

Variables	Category	*n* (%)
Usage of diagnostic modality	CT scan	99 (23.46)
	Cardiac catheterization	29 (6.87)
Postoperative issues	Residual lesions	149 (35.31)
	Right ventricular dysfunction	31 (7.35)
	Left ventricular dysfunction	26 (6.16)
	Biventricular dysfunction	15 (3.55)
	Redo surgery	22 (5.21)
	Pulmonary artery/arterial hypertension	129 (30.57)
Outcome	Discharged	406 (96.21)
	Death	16 (3.79)

**Table 4:** Operative procedures and their frequencies.

Name of operation	Numbers	Name of operation	Numbers
VSD closure	64	Aortic valve repair	03
ICR for TOF	47	Aortic valve replacement	03
PDA ligation	47	MAPCAs ligation/veno-venous collateral ligation	03
ASD closure	45	Modified Senning's operation	03
VSD closure + PDA ligation	28	Rheumatic heart disease (RHD)	03
Glenn shunt for single ventricle (SV) physiology	27	Arterial switch operation	02
VSD closure + ASD closure	18	Aortopulmonary window repair	02
Atrial septectomy for SV physiology	18	Atrial septation	02
PA banding	18	Brock’s operation	02
Mitral valve repair	17	Isolated left PAPVC re-routing	02
MPA ligation/tightening	16	Mitral valve replacement	02
Multiple VSDs closure	16	MPA plasty	02
BT shunt for TOF	15	Relief of RVOT	02
VSD closure + ASD closure + PDA ligation	15	Takeuchi operation for ALCAPA	01
Intra-ventricular tunnel repair (IVTR) for DORV	14	Truncus repair	02
RVOT resection	11	Sub-aortic membrane (SAM) excision	02
Sinus venosus SVC type ASD + PAPVC re-routing	11	Bilateral branch PAs plasty	01
Re-do sternotomy	10	Branch PAs reduction plasty	01
TAPVC repair	10	Coronary – cameral fistula ligation	01
Coarctation of aorta	08	Cone reconstruction of tricuspid valve	01
Complete AV canal repair	07	Classic/conventional repair for c-TGA	01
ASD closure + PDA ligation	06	Complete heart block	01
Pulmonary valvotomy	06	Confluence of pulmonary artery (PA) creation	01
Completion Fontan	05	Interrupted aortic arch (IAA) repair	01
Kawashima repair	05	Infundibulectomy	01
LPA plasty	05	Hypoplastic aortic arch repair	01
Cortriatriatum excision	04	PA de-banding	01
Interrupted IVC	04	Transitional AV canal repair	01
PDA division and suturing	04	Unifocalization of MAPCAs	01
Scimitar syndrome	04	Cardiac CT dynamic study	24
Tricuspid valve repair	04	Noncardiac operations/procedures	10

**Table 5:** Mortality by operative procedure (*n* = 16).

Name of operation	Numbers	Name of operation	Numbers
Ventricular septal defect closure	5	Redo sternotomy	1
Glenn shunt for single Ventricle Physiology	2	Relief of right ventricular outflow tract	1
Intracardiac repair for tetralogy of Fallot	2	Total anomalous pulmonary venous connection repair	1
Modified Senning's operation	1	Tricuspid valve repair	1
Pulmonary artery banding	1	Rheumatic heart disease	1

**Table 6:** Association of age, gender, and symptoms to the outcome.

Category	Subcategory	Discharged	Death	*p*-value*
Age (in months)	**0–12**	227	6	0.350
	**13–60**	86	5	
	**61–120**	61	2	
	**>120**	33	3	
Gender	**Male**	248	14	**0**.**011**
	**Female**	159	1	
Symptoms	**Symptomatic**	79	12	**0**.**001**
	**Not much symptomatic**	328	3	

* *p*-value based on Chi-square test.

Bold values signify the statistical significance.

**Table 7:** Association of postoperative issues with the type of cardiac defects.

Postoperative issues		Single or isolated acyanotic defects (138)	Other acyanotic CHDs^a^ (140)	Tetralogy of Fallot (68)	Complex cyanotic CHDs^a^ (65)	*p*-value*
Right ventricular dysfunction	Yes	14	10	6	1	0.146
	No	124	130	62	64	
Left ventricular dysfunction	Yes	17	7	1	1	**0** **.** **004**
	No	121	133	67	64	
Biventricular dysfunction	Yes	8	5	1	1	0.412
	No	130	135	67	64	
Redo surgery	Yes	9	4	2	7	0.097
	No	129	136	66	58	

* *p*-value based on Fisher exact test.

^a^ Congenital heart defects.

Bold values signify the statistical significance.

## Discussion

In this retrospective research, we analyzed the profile of CHDs and the postoperative course of CHD operated at our center, the only referral hospital in the central region of Gujarat operating pediatric cardiac patients. We compared the demographic details and pattern of CHD with similar studies conducted in India. In the current study, acyanotic and cyanotic cardiac defects accounted for 278 (68.6%) and 125 (31.4%), respectively, wherein the most common defect was ventricular septal defect (VSD)—21.84% (88/403) among acyanotic and tetralogy of Fallot's (TOF) and 14.89% (60/403) among cyanotic CHD variety. In all other studies, a similar proportion was noted (7–20). Around two-thirds of the burden was from acyanotic and one-third from cyanotic whereas VSD was the single-highest defect among acyanotic variety and TOF among the cyanotic variety. The present study has male sex dominance, 1.6:1. However, in one of the earlier studies from India, Khalil et al. did not find any difference in the male–female ratio when the diagnosis of CHDs was attempted at the time of birth to 72 h. However, Smita et al. showed male predominance when diagnosed in the first year of life and then no such trend in the difference of sex (7, 8). Other studies also reported variable male dominance ranging from 1.1:1 to 1.65:1 (9, 12–14, 17–20). If we look at age group-wise distribution, different studies have different distributions, especially in the less than 1-year age group, and variation ranging from 75%–80% to 37%–57% (8, 12, 13, 16, 17, 19, 20). The present study enrolled 233 (55%) infants. The difference can be explained by the study settings. In the current study, the median (Q1, Q3) age in months was 12 (6, 60) which was comparatively less than documented in the previous study (21). According to their findings, the average age at which echocardiography confirmed a CHD diagnosis was 18.6 months. Those with a higher study population of the infantile age group had a cardiothoracic or cardiology department as a referral center.

According to one study, chromosomal abnormalities (7% Down syndrome, 2% trisomy 18, and 1% trisomy 13) were linked to 12% of CHD occurrences, with an average overall frequency of 0.97 per 1,000 births (22), but we had only 3.08% syndromic patients. According to Khalil et al., 17.9% of CHD patients had somatic abnormalities. The most prevalent anomaly (9.3%, *n* = 4) was Down syndrome (7). As reported by Singh et al., 3% of patients with CHDs have Down syndrome (20). In our cohort, there was a lack of detailed evaluation and genetic confirmation of infants that might have culminated in fewer syndromic cases. In the present study, nearly 106 (25%) had one or more significant symptoms, but others with minimal or non-specific symptoms were diagnosed using 2D echocardiography. However, one of the studies emphasized that to fully comprehend the morphologic characteristics of CHD and their normal anatomy, an optimal computed tomography (CT) technique is to be chosen to achieve an accurate diagnosis (23). Failure to gain weight/poor weight gain, recurrent respiratory tract infection, chest pain or dyspnea on exertion, cyanotic spells, etc., were the main clinical features in the present study cohort (Table 1). One of the earliest Indian studies reported cyanosis in five (11.6%) of CHD cases on the first day of life only, whereas murmur (27.9%, *n* = 12) and cyanosis (16.2%, *n* = 7) were present during the initial week of life. The percentage of CHD cases with a murmur found after a week rose to 34.9%. The study showed that 119 (59.2%) were treated medically, but the remaining 210 patients required surgical management (20). Thirty-six (17.9%) patients had mortality throughout the 1-year follow-up period. However, in the present study, mortality was 16 (4.1%) deaths among all operated cases of 386. The period we studied also falls partially under the COVID-19 pandemic, but not a single death due to that. Sachdeva et al. showed that children who have heart disease are more likely to die if they contract COVID-19 (24).

Our center's in-hospital mortality rate of 3.9% is lower than the 5%–9% reported by other Indian tertiary centers (24–26) and is comparable to the Hridyam program's 2.4% mortality in Kerala (27). This may be due to our case mix, with a predominance of lower-risk acyanotic defects (68.6%) and fewer complex cyanotic cases requiring advanced support like ECMO, which are referred. Consistency from a single surgeon and dedicated pediatric cardiac ICU likely contributed to improved outcomes. These results highlight that even a semi-urban center can achieve outcomes like national benchmarks with streamlined care pathways.

In the present study, the most performed operations/procedures were VSD closure, intracardiac repair (ICR) for TOF, ASD closure, and PDA ligation, the same as those reported in the Hridyam patient profile of infants beyond the newborn period. The Hridyam model of Kerala gave hope for a better future for patients with CHDs by proving that their model worked to a great extent and contributed a lot by decreasing the infant mortality rate (IMR) to 7% from 12% from years 2016 to 2019 (27) which was the not the situation earlier as per the year 2008 report (25). The same author claimed in their universal heart coverage report a few years later that India lacked the necessary facilities and that the existing centers were not dispersed according to local needs. They predicted that challenges would remain for quite a long time in delivering quality care for heart defects in children (26). Furthermore, in the Hridyam patient profile, arterial switch, PDA stent, and total anomalous pulmonary venous connection (TAPVC) repair were the most frequent procedures performed in patients less than 1 month of age, whereas ASD device, ASD surgical closure, and PDA device were the most common procedures in patients above 1 year of age (27).

The Hridyam model, developed in Kerala, stands out as a pioneering statewide program that integrated clinical care with public health infrastructure to reduce congenital heart disease (CHD)-related mortality. Its novel features include a centralized, web-based case registration and tracking system that enables early detection, risk stratification, referral prioritization, and long-term follow-up of all children diagnosed with CHD. Hridyam connects primary health centers, district hospitals, and tertiary cardiac centers through a digital platform, ensuring that each child is followed in real time from diagnosis to surgery. In addition, the program utilizes public–private partnerships to subsidize or fully fund surgeries, contributing to a significant reduction in CHD-related infant mortality rates in Kerala (27). While our center operates outside a formalized statewide system like Hridyam, we have independently adopted several core principles that align with its goals. These include a streamlined perioperative workflow, multidisciplinary collaboration among pediatric cardiologists, intensivists, and surgeons, and standardized clinical pathways for acyanotic and cyanotic CHD. Our ICU provides 24/7 specialist coverage, and our center has the advantage of surgical continuity, with a single experienced cardiothoracic surgeon performing all procedures during the study period—likely contributing to low perioperative mortality (25, 27).

Unlike Hridyam's web-enabled statewide registry, our center relies on conventional referral systems without a digital case-tracking mechanism linking primary and secondary care facilities to our tertiary center. Additionally, our center serves as a regional referral hub within Gujarat but lacks the formalized network integration across multiple healthcare tiers that Hridyam facilitates. We also selectively refer the most complex cases—particularly those requiring extracorporeal membrane oxygenation (ECMO)—to higher-tier centers, unlike Hridyam's model, which included protocols for the systematic distribution of complex cases across multiple specialized cardiac centers.

If operative and postoperative periods are discussed, it is known that only one-fourth received optimal cardiac care, as stated by Anita Saxena in her 2018 report. (1) Over a year (2016–2017), almost 27,000 people with CHD had heart surgery. Only 25% of live babies receive the necessary cardiac care, despite the typical birth rate of CHDs requiring intervention in infancy being 1.6/1,000. (1) Although this percentage is still less, it is far better than comparable estimates from India published by the same author ten years ago. (2) The present study reported around 100 surgeries per year, and due to a lack of data or registry of cardiac defects, it is impossible to compare how many received optimal care among serious prevalent CHDs in our region. According to Zacharias's study, children with congenital heart disease had a 17.7 (95% CI: 16.8–18.6) times higher death risk than controls. Patients with CHD have the highest mortality risk throughout their first 4 years of life (28). However, we are reporting 4.1% of deaths among all patients who have operated at our center, which is slightly higher than those reported by the Kerala state of India in recent times (6). They reported having 2.4% postoperatively. However, 5% preoperative mortality was also reported due to the lack of universal catchment of all cases (29). According to an adult study, one of the main issues with the long-term follow-up of adults with CHD following heart surgery is heart failure (HF). Right ventricular performance, age, frequency of reoperations, and diagnosis all play a significant role (30). In the current study, ∼35% had residual lesions, 17% had ventricular dysfunction, and 5.2% had redo surgeries as major postoperative issues. Around 30% had pulmonary hypertension at the time of discharge. Although a formal cause-of-death audit was not performed, ventricular dysfunction and residual lesions were present in most fatal cases, suggesting low cardiac output and postoperative hemodynamic compromise as likely contributors to mortality. In the Boston study, over 50% of mortality was attributed to low cardiac output rather than solely to ventricular failure. The study revealed considerable anatomical variation among patients who succumbed. Most patients (*n* = 79) were in the intensive care unit (ICU) before surgery, with seven fatalities occurring in the operating room. Among the 100 deaths, 73 patients had technically adequate surgical procedures, 23 had residual anatomical defects, and 4 had an indeterminate status. Causes of death included low cardiac output in 52, inadequate postoperative physiology in 24, ventricular failure in 19, pulmonary hypertension in 8, and valvular regurgitation in 1 case. Additionally, sudden cardiac arrest, sepsis, and procedural complications were responsible for 11, 11, and 8 cases, respectively (30). In their study, Zacharias et al. found that patients with non-conotruncal defects, such as endocardial cushion defects, common ventricle, and hypoplastic left heart syndrome, had the highest relative risk of mortality (28). They reported that CHD babies born in 2000 showed improved survival compared to the 1980s and 1990s, but no difference was noted between the two cohorts born in 2000 and 2010. No significant difference in survival trends was observed between male and female patients with CHD.

### Limitations

The present study provides valuable insights into pediatric heart surgeries at a semi-urban center in Gujarat. There are several limitations also. Its retrospective design restricts control over data collection, potentially leading to incomplete or inconsistent records that may affect reliability. Conducted in a single tertiary care center, the results may not be fully generalizable to other regions with varying healthcare resources and patient populations, which could limit external validity. Although the study focuses on immediate postoperative outcomes, it does not address long-term complications, re-hospitalizations, or survival rates, which can affect surgical outcomes. Additionally, without a control group or comparison with non-surgical cases, the effectiveness of the interventions cannot be assessed comprehensively. While most information was accessible through the file transfer protocol (FTP) system, reliance on hospital records alone might have introduced some data gaps. Lastly, the study does not include socioeconomic or environmental factors, which could provide a broader context on the challenges and outcomes for children with congenital heart disease in semi-urban and rural settings. Despite these limitations, the study contributes significant data from a semi-urban region, highlighting the capacity of centers outside major cities to manage complex pediatric cardiac cases. The main strength of the study is the significant number of patients operated on at our single center with comparable results despite resource constraints like the facility of extracorporeal membrane oxygenation (ECMO) and state-of-the-art heart–lung machine.

## Conclusion

Our regional cardiac center demonstrates the capability to provide high-quality surgical care for pediatric patients with a broad spectrum of cardiac defects, achieving outcomes comparable to those of larger centers.

## Data Availability

The datasets presented in this study can be found in online repositories. The names of the repository/repositories and accession number(s) can be found in the article/Supplementary Material.
